# The Institutional Library

**Published:** 1913-05-10

**Authors:** 


### THE INSTITUTIONAL LIBRARY.

Digestion and Diet. By Thomas Dutton, M.D. (The Walter Scott Publishing Co. Fourth Edition. 1913. Pp. 131. Price 2s. net.)

This book contains much useful information on the important subject of diet; but after reading it through one does not feel that one has got much "forrader." It has two other unfortunate drawbacks. One is the strong vein of egotism which pervades it all through; and the other is the recommendation in the text of various patent foods whose manufacturers advertise at the beginning and end of the letterpress.

A Manual of Surgical Treatment. By Sir Watson Cheyne, Bart., C.B., and F. F. Burghard, M.S., F.R.C.S. New Edition, with the assistance of T. P. Legg, M.S., F.R.C.S., and Arthur Edmunds, M.S., F.R.C.S. Vol. IV. (Longmans, Green and Co. 1913. Pp. 622. Price 21s. net.)

The completion of this splendid manual goes steadily forward, and in the present volume the authors treat of the surgical affections of the jaws, tongue, and alimentary canal. This involves, as will readily be appreciated, that extensive field of gastro-intestinal surgery in which such immense strides have been made since the original edition of the work. Thanks to the extremely businesslike way in which the root of every subject is tackled straight away without the slightest unnecessary verbiage, this enormous branch of surgical treatment has been successfully dealt with in little more than six hundred pages. He who would trace out ætiological factors to their remotest ramification, or seek detailed statistics concerning the merits of various operations, will not find them here; but the practitioner-surgeon is supplied with exactly what he wants as to diagnosis and treatment of the conditions which he meets in practice. Speaking generally, all that is progressive, yet safe and sane, in modern surgical teaching and technique is included.

In relation to a large and complicated subject such as this, differences of opinion are bound to exist. For our own part, we think the authors underrate the value of *x*-ray examination of bismuth meals in the diagnosis of gastric diseases. In the chapter on diagnostic methods they dismiss it rather contemptuously in eight lines, compared with five pages allotted to the administration and examination of test meals. Dawbarn's silk-thread method of diagnosis is also not mentioned. We note with interest that the clamp and cautery are advocated for piles, and the ligature method condemned. Another small point is the preference for chloroform or intravenous ether over ordinary (open) ether anæsthesia for gastro-enterostomy. The arguments for this recommendation do not convince us. Small points such as these, however, do not detract materially from our profound admiration for this excellent book.

Lessons on Elementary Hygiene and Sanitation, with Special Reference to the Tropics. By W. T. Prout, C.M.G., M.B. (London: J. and A. Churchill. Third Edition. Pp. 184. Price 2s. 6d. net.)

These lessons were apparently originally written for an educated negro audience in West Africa; but we do not doubt that they have proved a blessing also to European inhabitants of the once dreaded West Coast. It is to Dr. Prout and his former colleagues of the West African Medical Service that the declining reputation of those regions as a white man's grave is due. And since prevention is better than cure, the hygienist's work is even more important and beneficial than the healer's. We are not surprised that a third edition of this book has been required. We would pack a copy of it in the kit of every "new chum" proceeding to West Africa or any other part of the tropics; and for the matter of that in every old one's too.

Right Diet for Children. By Edgar J. Saxon. (London: C. W. Daniel, Ltd. 1913. Pp. 89. Price 1s.)

In this interesting little work the author deals with the feeding of growing children on non-flesh lines. He indicates very lucidly the most common mistakes in feeding and their result, and in explaining the principles of his idea of correct diet he gives his readers several practical suggestions. The introductory chapter by Florence Daniel should prove exceptionally suggestive to those charged with the care of infants, for it is based upon sound common sense, and, notwithstanding the title of the work, contains no trace of dogmatism. The philosophy of the point of view expressed concerning health and disease appeals to the sense of beauty. Though the teaching must be regarded in many respects as a breach of orthodoxy, the work possesses undoubted merit, and the style is sufficiently popular to render it intelligible to the average observant layman.

